# Interrogation of Internal Workings in Microbial Community Assembly: Play a Game through a Behavioral Network?

**DOI:** 10.1128/mSystems.00550-19

**Published:** 2019-10-29

**Authors:** Qian Wang, Xinjuan Liu, Libo Jiang, Yige Cao, Xiang Zhan, Christopher H. Griffin, Rongling Wu

**Affiliations:** a Center for Computational Biology, College of Biological Sciences and Technology, Beijing Forestry University, Beijing, China; b Department of Gastroenterology, Beijing Chao-Yang Hospital, Capital Medical University, Beijing, China; c Department of Public Health Sciences, Penn State Hershey College of Medicine, Hershey, Pennsylvania, USA; d Applied Research Laboratory, The Pennsylvania State University, University Park, Pennsylvania, USA; University of California San Diego

**Keywords:** game theory, competition, cooperation, microbial interaction network

## Abstract

Increasing evidence shows that the influence of microbiota on biogeochemical cycling, plant development, and human health is executed through a complex network of microbe-microbe interactions. However, characterizing how microbes interact and work together within closely packed and highly heterogeneous microbial ecosystems is extremely challenging. Here, we describe a rule-of-thumb framework for visualizing polymicrobial interactions and extracting general principles that underlie microbial communities. We integrate elements of metabolic ecology, behavioral ecology, and game theory to quantify the interactive strategies by which microbes at any taxonomic level compete for resources and cooperate symbiotically with each other to form and stabilize ecological communities. We show how the framework can chart an omnidirectional landscape of microbial cooperation and competition that may drive various natural processes. This framework can be implemented into genome-wide association studies to unravel the genetic mechanisms underlying microbial interaction networks and their evolutionary consequences along spatiotemporal gradients.

**IMPORTANCE** Identifying general biological rules that underlie the complexity and heterogeneity of microbial communities has proven to be highly challenging. We present a rule-of-thumb framework for studying and characterizing how microbes interact with each other across different taxa to determine community behavior and dynamics. This framework is computationally simple but conceptually meaningful, and it can provide a starting point to generate novel biological hypotheses about microbial interactions and explore internal workings of microbial community assembly in depth.

## Abstract (precis)

Identifying general biological rules that underlie the complexity and heterogeneity of microbial communities has proven to be highly challenging. We present a rule-of-thumb framework for studying and characterizing how microbes interact with each other across different taxa to determine community behavior and dynamics. This framework is computationally simple but conceptually meaningful, and it can provide a starting point to generate novel biological hypotheses about microbial interactions and explore internal workings of microbial community assembly in depth.

## INTRODUCTION

Microbial communities affect a wide range of natural processes, from biogeochemical cycling to plant development and animal and human health (1, 2). Surveys of microbiome composition across various ecological settings from the soil to the human body have consistently revealed that microbes are organized into functional and stable communities through fundamental ecological principles (3, 4). However, the manner in which the structural-functional relationship of polymicrobial communities is established remains poorly understood, largely because we know little about the ways in which microbes interact with each other.

Different microbes in the same community would compete for resources and space but also cooperate through metabolic exchange or quorum sensing to reach the community’s equilibrium (5, 6). This process proceeds like a game. Game theory, originally developed in economic research (7), enables the formulation of an individual strategy that maximizes payoff by incorporating the strategies of other members (8). Several authors have used pairwise game theory to study the structure of microbial communities (9–11). However, in microbial community assembly as a densely packed ecosystem, one microbe may interact not merely with a single member but rather with multiple members to form a complex network. Recent attempts have been made to elucidate the architecture of microbial interactions using network tools (12–17), but these tools need difficult-to-collect longitudinal abundance data to infer informative microbial networks.

Here, we build a quantitative framework for interrogating and interpreting the pattern and distribution of microbial interactions within microbial community assembly. We expand and scale up game theory to large, complex network systems through a simplified mathematical formulation. Not relying on the availability of longitudinal data, our network game framework constructs microbial networks of any dimension and at any level of phylogenetic taxa. The quantitative feature of the framework enables it to identify and predict the general principles that modulate the alterations of microbial interactions.

## RESULTS

### Integrating metabolic theory and game theory into microbial networks.

Metabolic theory states that the physiological, morphological, and life history traits of an organism vary with its size among individuals or species in the power law (18, 19). This theory characterizes a phenomenon that is widespread at all levels of organization from individuals to the biosphere. Here, we use a power equation to describe how the capacity of a particular microbe to survive and proliferate, broadly defined as fitness (*F*), scales with its abundance (*N*), expressed as

(1) $$F=N_{0}N^{b},\text{ or}\;F/N_{0}=N^{b}$$

where *N*_0_ is a normalized constant and *b* is the scaling exponent. By taking the logarithm of the two sides of equation 1 and moving *b* to the left side, we obtain

(2) $$\text{log(}F\text{/}N_{0}\text{)/}b=\text{ log}\;N$$

Here, we define $G=\text{ log(}F\text{/}N_{0}\text{)}/b$ as the fitness index of the microbe. The equality of equation 2 implies that the abundance *N* can be used as a proxy of the fitness of the microbe.

**FIG 1:**
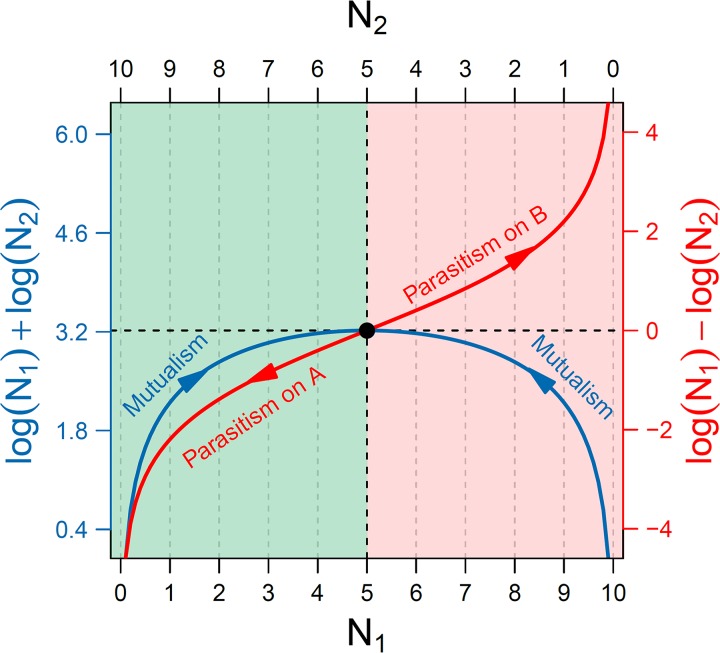
Quantitative description of mutualism (blue) and parasitism (red). Assume a microbial community in which two microbes A and B, whose abundance is denoted by *N*_1_ and *N*_2_, respectively, interact with each other. The blue curve, specified by log *N*_1_ + log *N*_2_, describes how the two microbes cooperate to form mutualism, with arrows indicating the increase of mutualism with increasing equality of their abundance. The red curve, specified by log *N*_1_ – log *N*_2_, is indicative of parasitism through competition, by which one microbe gains by reducing the fitness of the other, with arrows representing the direction of increasing parasitism. The black circle is the position at which mutualism achieves a maximum degree whereas parasitism is minimum.

Consider a pairwise interaction as a game, in which two microbes, each as a player, tend to maximize their own payoff (fitness) through an action contingent upon the strategy of the counterpart. In a so-called zero-sum game, the interests of the players are in complete conflict; that is, one player’s gain is always another player’s loss. Complete cooperation implies that the two players achieve a maximum gain simultaneously. Let *G*_1_ and *G*_2_ denote the fitness indices of two microbes A and B, respectively, whose sum is positively correlated with the strength of their cooperation. This can be proven by the following expression for the “inclusive” fitness of the two microbes:

(3) $$G_{\text{1}}+G_{\text{2}}=\text{log }N_{1}+\text{log }N_{2}=\text{log }N_{1}N_{2}$$

where *N*_1_ and *N*_2_ are the abundance levels of microbes A and B, respectively. Given the amount of resources shared by the two microbes, i.e., *N*_1_ + *N*_2_ is fixed, this sum (inclusive fitness) achieves a maximum value only when *N*_1_ is equal to *N*_2_, which implies that complete mutual cooperation (*N*_1_ = *N*_2_) can ensure that both microbes obtain a maximum fitness simultaneously and, therefore, a maximum inclusive fitness (Fig. 1). Any value below this maximum is due to deviation from the equality *N*_1_ = *N*_2_. As such, the log product of abundances of two microbes, log *N*_1_*N*_2_, can be used to measure and quantify the strength of their cooperation, i.e., the amount of mutualism or mutual benefits.

In microbial communities where resources become limited, a microbe will grow at the cost of its conspecific microorganism. The asymmetric form of such a competitive relationship is called predation or parasitism by which one microbe benefits by consuming and damaging the resources of others. We define the difference of fitness of two microbes as exclusive fitness to mathematically describe the strength of parasitism, expressed as

(4) $$G_{\text{1}}-G_{\text{2}}=\text{log }N_{1}-\text{log }N_{2}=\text{log(}N_{1}/N_{2})$$

where the growth of microbe A proceeds at the expense of microbe B, with the extent of such parasitism quantified by the log ratio of the abundances of two microbes, log (*N*_1_*/N*_2_). This ratio can be used as a measure for the parasitism of one microbe at the expense of the other, depending on whether it is larger or less than zero (Fig. 1). For consistency, we stipulate the larger abundance as the numerator and the smaller abundance as the denominator to calculate the ratio.

Plotting log products and log ratios of the abundance of two microbes in the same illustration allows the comparison of mutualism and parasitism as two distinct processes that govern the microbial community assembly (Fig. 1). The two microbes approach their highest peak of mutualism when their abundance levels, *N*_1_ and *N*_2_, tend to be equal, whereas parasitism becomes predation in the case in which one microbe completely inhibits the other, leading *N*_1_ and *N*_2_ to have opposite abundance levels. Between complete cooperation and complete competition are the intermediate processes that not only include cooperation but also implicate competition. In other words, cooperation may contain competition, while competition may also include cooperation. It is likely that cooperation and competition, two different but dynamically related processes, together underlie the overall microbial community behavior.

### Interpreting the mathematical descriptors of ecological interactions from behavioral ecology.

Equations 3 and 4 that quantitatively describe the strengths of mutualism and parasitism have an ecological basis. In behavioral ecology, collective motion phenomena, such as swarming, flocking, and schooling behavior, are regarded as being ubiquitous in a large variety of animal species ranging from bacteria to humans (20, 21). Under natural selection, such collective animal behavior has been shaped in two important ways. First, animals of roughly similar size in a population tend to cooperate with each other to form group-level coordination under rules of attraction and alignment (22–25). Animals prefer to shoal and cooperate with individuals that resemble themselves, because any shoal member that stands out in appearance will be preferentially targeted by predators, a phenomenon called the “oddity effect” (26). As such, if two animals cooperate mutually, they tend to be of similar body size. In mathematics, given that the sum of two variables is fixed, their product reaches a maximum when they have the same value. Thus, since the magnitude of the product is related to how much the two variables are similar in value, we hypothesize that the product of two animals’ body sizes is positively correlated with the strength of cooperation.

Second, dominant animals of large body size tend to be agonistic to subordinates of small body size during a conflict between members of the same population (27, 28). Such agonistic behavior, regarded as an adaptive aggressive and defensive action, plays a pivotal role in resource acquisition, reproductive success, and even survival (29). On the basis of the animal behavioral theory described above, we hypothesize that the ratio of body mass of a larger animal to a small animal in the socialized environment can serve as a surrogate of the strength of parasitism.

### Topological landscape of cooperation and competition: a true story from the gut microbiota.

**FIG 2:**
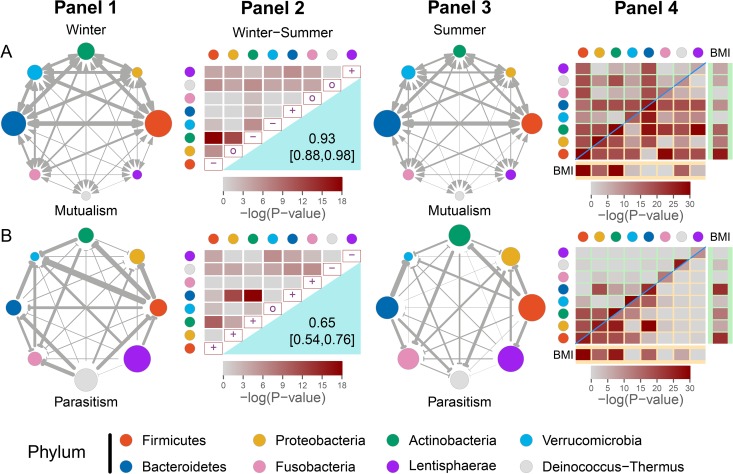
The network of mutualism (A) and parasitism (B) among eight phyla (distinguished by colors) derived from 127 hosts of the Hutterites, including 93 in winter and 91 in summer. (Panels 1 and 3) Mutualism (indicated by double arrowed lines) and parasitism (indicated by T-shaped lines) expressed independently in winter and summer. The size of the circles indicates the strength of mutualism or parasitism among different genera within phyla, whereas the thickness of the lines indicates the strength of pairwise mutualism or parasitism among different genera from different phyla. (Panels 2) On the diagonal of the matrix are the signs of within-phylum mutualism or parasitism changes from winter to summer (+ for increase, 0 for no change, and – for decrease). The right bottom part reports the correlation coefficient (with its estimated confidence interval) of mutualism or parasitism between the two seasons, representing the season-dependent similarity of the mutualism or parasitism networks. The left top part reports a colored scale representing the results of the significance test of the season-dependent strength difference of mutualism or parasitism between the same phylum pairs. The more intense the color, the higher the degree of mutualism or parasitism. (Panels 4) Cells on the diagonal of the matrix represent the significance tests of correlations of within-phylum mutualism (A) or parasitism (B) with BMI, each of which is separated into two parts for winter (left top) and summer (right bottom). The left top and right bottom portions represent the significance test of the correlations of between-phylum mutualism or parasitism with BMI. The significance tests of the correlations of the microbial abundance of each phylum with BMI are given in the BMI columns (winter) and the BMI rows (summer).

The gut of a healthy human contains symbiotic interactions among 500 to 1,000 bacterial species, belonging to different genera, all the way up through families, orders, classes to phyla (30). All these highly heterogeneous bacteria interact and work together to determine the stability of the microbial community and further impact human health. Davenport et al. (31) reported 16S rRNA gene sequencing data of the gut microbiota at 101 genera and 8 phyla collected from 127 hosts of a founder population, the Hutterites, of which 93 were sampled in winter, 91 in the following summer, and 57 in both winter and summer. We calculated pairwise log products of abundance between different genera and pairwise log ratios of abundance of a bigger genus over a smaller genus, which allowed us to reconstruct 93 winter-specific 101-node networks and 91 summer-specific 101-node networks for mutualism and parasitism, respectively. We merged higher-dimensional genus-level networks into lower-dimensional phylum-level networks by averaging log values over genera from within the same phylum (used as nodes) and those over genus pairs from different phylum pairs (used as edges). Averaged over all hosts from the same season, we reconstructed eight-node mutualism and parasitism networks for winter and summer, respectively (Fig. 2). The significance of mutualism and parasitism within and between phyla was tested using information about sampling variances among hosts. A detailed procedure of reconstructing and testing networks is given in Materials and Methods.

Strong mutualism and parasitism exist in the gut microbiota, but the extent and frequency of these interactions depend on the phylum considered and how phyla are paired and are also affected by seasonal change. Of all phyla, *Firmicutes* and *Bacteroidetes* not only display the most intense genus-genus mutualism within their own phyla but also pursue the most intense cooperation with one another and other phyla as well (Fig. 2A). It seems that the mutualism involving these two phyla dominate the network of mutualism. Many phyla were found to have strong internal competition, although such within-phylum competition mostly increases from winter to the next summer (Fig. 2B). In general, mutualism between pairwise phyla is strongly correlated between winter and summer (*r *= 0.93), with a larger extent than the correlation of competition between the two seasons (*r *= 0. 65). Significance tests discerned different patterns of how the strengths of mutualism and parasitism vary with season. From winter to summer, within-phylum mutualism decreases for *Firmicutes* and increases for *Bacteroidetes*, but it is stable for the other phyla, whereas between-phylum mutualism generally decreases (Fig. 2A). The strength of mutualism involving *Firmicutes*, *Actinobacteria*, and *Proteobacteria* decreases dramatically from winter to summer (*P* < 10^−9^). *Lentisphaerae* and *Deinococcus*-*Thermus* are the two phyla that exhibit weak internal and weak external cooperation with other phyla, with a slight season-dependent change.

From winter to summer, within-phylum parasitism increases for most phyla, including *Firmicutes* and *Bacteroidetes*, decreases for *Deinococcus*-*Thermus* and *Lentisphaerae*, and is unchanged for *Verrucomicrobia*. Figure 2B shows the topological network of directed and weighted between-phylum parasitism. Both *Firmicutes* and *Bacteroidetes* tend to parasitize other phyla, such as *Actinobacteria*, *Proteobacteria*, and *Fusobacteria*, but are parasitized by *Verrucomicrobia* in the two seasons. It is interesting to see that *Firmicutes* parasitizes *Bacteroidetes* in winter, but this relationship changes during summer. All these above parasitic relationships construct the main sketch of the network. In general, the strength of parasitism between phyla decreases from winter to summer, and this phenomenon may be due to the necessity in winter to favor microbes allowing for higher digestion and absorption of nutrients to store more energy for the cold season as well as to the fact that summer offers a greater nutritional variety which *per se* enhances a greater microbial diversity compared to winter.

Our theory can estimate and test the correlations of microbial cooperation and competition with host health risk. The plot of correlation significance tests shows how mutualism or parasitism covaries with the health risk trait of body mass index (BMI) over sampled hosts. Considering all possible pairs, mutualism has a much larger likelihood to correlate with BMI than parasitism (Fig. 2). In general, the correlations of both mutualism and parasitism with BMI are largely consistent over season, except for a few cases. Mutualism within phyla is highly correlated with BMI, especially for some phyla such as *Firmicutes*, *Bacteroidetes*, and *Actinobacteria*, and parasitism within phyla is also highly correlated with BMI, but with some of these correlations changing over seasons. A strong correlation of within-phylum parasitism with BMI for *Deinococcus*-*Thermus* occurs in winter, but it decreases dramatically in summer. There is a particular group of phylum pairs that are correlated with BMI through mutualism or parasitism. For example, *Firmicutes* and *Bacteroidetes* each cooperate with all other phyla to relate to BMI (Fig. 2A). *Actinobacteria* cooperates with more phyla in summer than in winter to link to BMI. The parasitism of *Firmicutes* negatively impacting *Actinobacteria* and *Proteobacteria* is remarkably correlated with BMI in both seasons (Fig. 2B). A similar case is true for the parasitism of *Proteobacteria* affecting *Bacteroidetes* and *Actinobacteria*. *Bacteroidetes* parasitizes *Verrucomicrobia*, and this interaction is linked to BMI in summer, but not in winter.

In previous studies, the microbiota impact on host performance was assessed by the correlation analysis of microbial abundance with a host trait (31). However, if we incorporate microbial interactions into such a correlation analysis, we can make new discoveries. For example, within-phylum mutualism for *Bacteroidetes* is more strongly correlated with BMI than *Bacteroidetes* microbial abundance in both seasons, especially in summer (Fig. 2A). A similar phenomenon was observed for other phyla, such as *Verrucomicrobia*. Also, parasitism is associated with BMI in a different way than microbial abundance is. For instance, within-phylum parasitism for *Verrucomicrobia* exhibits much stronger correlations with BMI than its abundance in both winter and summer (Fig. 2B). All these data suggest that our theory provides a new view of the influence of microbiota, thanks to dissecting how different microbes interact with each other in a topological network.

### Transition from cooperation to competition.

**FIG 3:**
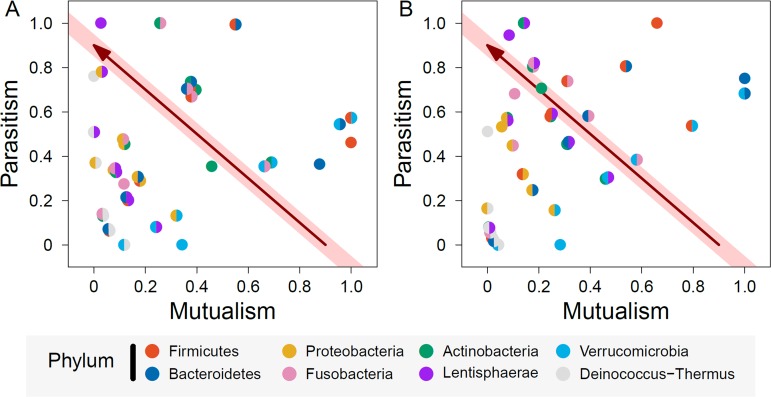
Correlations between mutualism and parasitism over phylum pairs in winter (A) and summer (B). Single-colored and double-colored circles are within-phylum and between-phylum mutualism or parasitism, respectively. The arrowed line on the diagonal shaded area indicates the change of microbial interaction from maximum mutualism to maximum parasitism.

There are many biological processes at all levels of organization, where cooperation intertwines with competition (32, 33). In a microbial system like the one shown in Fig. 1, two microbes may strive to cooperate until they achieve a complete level of cooperation. Any incomplete cooperation accompanies competition by which two microbes hinder each other’s fitness. Again, using Davenport et al.’s (31) gut microbiota data, we standardized the values of log products and log ratios among 8 phyla and 28 phylum pairs and plotted standardized log ratios (parasitism) against standardized log products (mutualism) in winter and summer, independently (Fig. 3). The results show that mutualism and parasitism are independent of one another, and therefore, these two interaction states may mutually and reciprocally transit from one state to the other over seasons.

By linking maximum mutualism to maximum parasitism by a line, we can characterize how a particular microbe transits from cooperation to competition. The microbes located on or near this line are thought to be in a cooperation-competition steady state. Microbes below this line tend to display milder interactions, whereas those above this line exert more intense interactions. Genera within *Actinobacteria* trigger moderate strengths of mutualism and parasitism in winter (Fig. 3A), but they are characterized by stronger parasitism and weaker mutualism during summer (Fig. 3B). In general, for some phyla, such as *Verrucomicrobia*, the relative importance of mutualism and parasitism is stable between the two seasons, but for many phyla and phylum pairs, the relative importance of mutualism and parasitism changes dramatically from winter to summer. A quantitative assessment on how each phylum or phylum pair is apt to cooperate or compete can be seen from Fig. 3, along with its season-dependent change.

## DISCUSSION

Of the ecological forces that govern the assembly and stability of microbial communities, microbial interactions have proven to be extremely difficult to study because of their high complexity, high heterogeneity, and high dynamics. Although temporal or perturbed data are powerful for inferring microbial networks, they are usually unavailable and expensive (34). As such, a simple, or even rough, approach that can capture main information about microbial interaction networks from high-dimensional microbial consortia becomes highly essential. Results from such an analysis can serve as a starting point to investigate how microbes interact with each other to determine community phenotypes at high resolution. By integrating elements of metabolic ecology, behavioral ecology, and game theory, we proposed a rule-of-thumb framework for detecting, testing, and cataloguing microbial interactions. This network game model enables the reconstruction of microbial networks at any dimension for microbial community assembly with any density and heterogeneity.

Our model found that the greatest amount of mutualism and parasitism occurs within and between the *Bacteroidetes* and *Firmicutes* phyla. Using more informative longitudinal abundance data from the gut microbiota, Venturelli et al. (34) identified the critical role of microbial interactions exerted by these two phyla in shaping microbial communities. The consistency of our result with the results from a well-designed experiment suggests the biological relevance of our model. Interactions may occur between different microbes from the same taxon and different taxa. It is also possible that interactions take place across phylogenetic clades. For example, one microbe from a taxon may interact with the entire group of other higher taxa. In Materials and Methods, we describe a full model to characterize microbial interactions at all possible levels of phylogeny. There is some evidence that intraspecific interactions can influence interspecific interactions (5, 17). Similarly, interspecific interactions may affect interactions between different genera, and intergenus interactions may influence interactions between different families, etc. These complex hierarchical patterns of interactions across clades can be identified using a statistical testing procedure outlined in Materials and Methods (Tables 1 and 2).

**TABLE 1:** Data structure of microbial abundance from different strains, species, genera, families, orders, classes, and phyla

Phylum	Class	Order	Family	Genus	Species	Strain	Abundance
1	1	1	1	1	1	1	*y*_1111111_
1	1	1	1	1	1	2	*y*_1111112_
1	1	1	1	1	2	3	*y*_1111123_
1	1	1	1	1	2	4	*y*_1111124_
1	1	1	1	2	3	5	*y*_1111235_
1	1	1	1	2	3	6	*y*_1111236_
1	1	1	1	2	4	7	*y*_1111247_
1	1	1	1	2	4	8	*y*_1111248_
⫶	⫶	⫶	⫶	⫶	⫶	⫶	⫶
*O*	$N=\overset{M}{\underset{m=1}{\sum }}N_{m}$	$M=\overset{N}{\underset{n=1}{\sum }}M_{n}$	$L=\overset{M}{\underset{m=1}{\sum }}L_{m}$	$K=\overset{L}{\underset{l=1}{\sum }}K_{l}$	$J=\overset{K}{\underset{k=1}{\sum }}J_{k}$	$I=\overset{J}{\underset{j=1}{\sum }}I_{j}$	*y_IJKLMNO_*
							

**TABLE 2:** Reorganized data structure by pairing all strains from different species, genera, families, orders, classes and phyla

Pair	Strain	Phylum	Class	Order	Family	Genus	Species	Undirected mutualism	Directed parasitism
1	1111111 × 1111112	1 × 1	1 × 1	1 × 1	1 × 1	1 × 1	1 × 1	$y_{1}^{+}$ = log *y*_1111111_ + log *y*_1111112_	$y_{1}^{-}$ = log *y*_1111111_ – log *y*_1111112_
2	1111111 × 1111123	1 × 1	1 × 1	1 × 1	1 × 1	1 × 1	1 × 2	$y_{2}^{+}$ = log *y*_1111111_ + log *y*_1111123_	$y_{2}^{-}$ = log *y*_1111111_ – log *y*_1111123_
3	1111111 × 1111124	1 × 1	1 × 1	1 × 1	1 × 1	1 × 1	1 × 2	$y_{3}^{+}$ = log *y*_1111111_ + log*y*_1111124_	$y_{3}^{-}$ = log*y*_1111111_ – log *y*_1111124_
4	1111111 × 1111235	1 × 1	1 × 1	1 × 1	1 × 1	1 × 2	1 × 3	$y_{4}^{+}$ = log *y*_1111111_ + log *y*_1111235_	$y_{4}^{-}$ = log*y*_1111111_ – log *y*_1111235_
5	1111111 × 1111236	1 × 1	1 × 1	1 × 1	1 × 1	1 × 2	1 × 3	$y_{5}^{+}$ = log *y*_1111111_ + log*y*_1111236_	$y_{5}^{-}$ = log*y*_1111111_ – log *y*_1111236_
6	1111111 × 1111247	1 × 1	1 × 1	1 × 1	1 × 1	1 × 2	1 × 4	$y_{6}^{+}$ = log *y*_1111111_ + log *y*_1111247_	$y_{6}^{-}$ = log *y*_1111111_ – log*y*_1111247_
7	1111111 × 1111248	1 × 1	1 × 1	1 × 1	1 × 1	1 × 2	1 × 4	$y_{7}^{+}$ = log *y*_1111111_ + log*y*_1111248_	$y_{7}^{-}$ = log *y*_1111111_ – log *y*_1111248_
⫶	⫶	⫶	⫶	⫶	⫶	⫶	⫶	⫶	⫶
*W*	(*O*–1) (*N*–1) (*M*–1) (*L*–1) (*K*–1) (*J*–1) (*I*–1) × *ONMLKJI*	*O*–1×*O*	*N*–1×*N*	*M*–1×*M*	*L*–1×*L*	*K*–1×*K*	*J*–1×*J*	$y_{W}^{+}$ = log *y*(*O*–1) (*N*–1) (*M*–1) (*L*–1) (*K*–1) (*J*–1) (*I*–1) + log *y_ONMLKJI_*	$y_{W}^{-}$ = log *y*(*O*–1) (*N*–1) (*M*–1) (*L*–1) (*K*–1) (*J*–1) (*I*–1) – log *y_ONMLKJI_*

Although we focus on modeling mutualism and parasitism, our model can be quantitatively generalized to study other types of microbial interactions, such as antagonism (by which two microbes compete against each other), commensalism (where one microbe benefits the second, but the latter has no effect on the former), and ammensalism (in which one microbe harms the second, whereas the second microbe has no effect on the first). Because the log product of abundance of two microbes is proportional to the strength of mutualism, its value may span a range from cooperation to competition. Similarly, the magnitude of the log ratio of abundance of a bigger microbe to a smaller microbe reflects the strength of parasitism, which covers commensal or ammensal relationship. Yet, distinguishing between commensalism and ammensalism needs additional information.

It should be pointed out that our model was derived from the integration of multiple disciplines. Its biological interpretation is founded on the metabolic and behavioral ecology of animal body size, which allows us to reconstruct microbial networks from abundance data. However, predators discern a target from a group of prey not only based on prey size but also prey color or even prey smell (26). It is unclear how to incorporate color- or smell-based oddity effects into our game network model, which presents a topic of great interest deserving further investigation.

By quantifying the inner workings of microbial community assembly, our network game model overcomes the descriptive limitation of empirical approaches for describing the global behavior of microbial communities. The model can be merged with evolutionary studies to investigate the fitness consequences of ecological interactions along a spatiotemporal gradient helping to identify the reason why a particular pattern of microbial interactions is favored by natural selection. In host-microbe interaction studies, this model can find its application in unveiling the genetic and molecular mechanisms underlying microbial interactions and identify genes that play a key role in shaping microbial networks. It can further construct a causal or predictive link of microbial networks within host phenotypes. With these capacities, the model provides a tool to dissect and engineer the interactions within microbial communities.

## MATERIALS AND METHODS

### Estimating mutualistic and parasitic relationships.

Consider a microbial community in which ecological interactions occur at the lowest taxonomic level. Suppose that we use a high-resolution technique that allows constituent microbes to be classified at the strain level. Let *y_ijklmn_* denote the abundance of strain *i* from species *j* from genus *k* from family *l* from order *m*, class *n*, and phylum *o*, with *i *= 1, …, *I_j_*, *j *= 1, …, *J_k_*, *k *= 1, …, *K_l_*, *l *= 1, …, *L_m_*, *m *= 1, …, *M_n_*, *n *= 1, …, *N*, *o *= 1, …, *O*. To illustrate the idea of game theory integration, we use a didactic example with the data structure (Table 1), where abundance is shown at hierarchic levels of classification. We describe the mutualism and parasitism of two different strains by the product of the abundances of the two strains and the inverse of the product, respectively, and the reciprocal altruism of two strains by the ratio of the abundance of the two strains. By taking log transformation of these derivatives, we obtain measures of cooperation versus competition and reciprocal altruism by the summation and difference of two log-transformed abundances, respectively. To reflect these measures, we reorganize the data by pairing different strains in the format given in Table 2. Since the total number of strains is *I*, there are *W *= 1/2*I*(*I* – 1) pairs. Let $y_{w}^{+}$ and $y_{w}^{–}$ denote the summation and difference of abundance between two strains in pair *w* (*w *= 1, …, *W*), on which we can test the effect of species pairs. Species pairs have 1/2*J*(*J* – 1) possibilities. For the *s*th species pair, the number of strain pairs is denoted by *T_s_*.

### Testing mutualistic and parasitic interactions.

We formulate a multiplicative likelihood at the species pair level, expressed as

(5) $$L_{s}(y_{+},y_{-})=\overset{\frac{1}{2}(J-1)J}{\underset{s=1}{\prod }}\overset{T_{s}}{\underset{w=1}{\prod }}f_{s}(y_{w}^{+},y_{w}^{-})$$

where $f_{s}(y_{w}^{+},y_{w}^{-})$ is a bivariate normal density function of the summation and difference of abundance of strains in pair *w*, characterized by mean vector $\left({\text{μ}}_{s}^{+},{\text{μ}}_{s}^{-}\right)$ and the covariance matrix composed of variance of strain summation $\sigma_{+}^{2},$ variance of strain difference $\sigma_{-}^{2},$ and their correlation ρ.

After the parameters determining the density function are estimated, we can formulate the following hypotheses:

$$\begin{array}{l} {\text{H}}_{0}:({\text{μ}}_{s}^{+},{\text{μ}}_{s}^{-})\equiv ({\text{μ}}_{+},{\text{μ}}_{-})\\ {\text{H}}_{\text{1}}:({\text{μ}}_{s}^{+},{\text{μ}}_{s}^{-})\neq ({\text{μ}}_{+},{\text{μ}}_{-})\text{ for all }s=1,. . .,1/2J(J-1) \end{array}$$

The acceptance of H_1_ suggests that species pairs determine ecological interactions among different strains through the summation and difference of abundance between different strains. Furthermore, by testing H_0_: ${\text{μ}}_{s}^{+}\equiv {\text{μ}}_{+}$, we can characterize whether mutualism or parasitism exists among microbes at the species level. Similarly, the existence of reciprocal altruism can be characterized by testing H_0_: ${\text{μ}}_{s}^{-}\equiv {\text{μ}}_{-}.$

Similar procedures can be extended to test whether and how microbes interact at the other higher levels, such as genus, family, order, class, and phylum levels. More interesting, the model can test whether and how microbes from a lower level interact with those from a higher level. For example, a particular set of species may interact with the microbes at the order level. This can be tested by formulating a certain hypothesis.

### Calculating correlations.

The following correlations are calculated.

(1) Network correlation. How two networks differ can be assessed by calculating the correlations (*r*) of nodes and edges between the networks. We calculate *r* between mutualism networks from winter and summer and between parasitism networks from winter and summer. These *r* values allow us to compare how networks change from season to season.
(2) Interaction correlation. How microbial interactions at the phylum level change from winter to summer can be assessed by calculating the correlations of edges of genus-level networks across hosts from each season. These results are given in the upper part of winter-summer matrix in panels 2 (Fig. 2).
(3) Microbial abundance-host trait correlation. We calculated the correlations between microbial abundance at the phylum level and BMI across hosts from a different season. These results are given in the BMI columns and rows in panel 3 (Fig. 2).
(4) Microbial interaction-host trait correlation. We calculate the correlations between microbial interactions within and between phyla and BMI across hosts from a different season. The correlations between microbial interactions within and between phyla and BMI across hosts from a different season are given on the diagonal and top part of matrix, respectively, in panels 3 (Fig. 2).

### Data availability.

The data used can be downloaded at https://doi.org/10.1371/journal.pone.0140301. The computer code can be requested from the corresponding author.
